# Comparison of the Effect of Velvet Antler from Different Sections on Longitudinal Bone Growth of Adolescent Rats

**DOI:** 10.1155/2016/1927534

**Published:** 2016-06-13

**Authors:** Hye Kyung Kim, Myung-Gyou Kim, Kang-Hyun Leem

**Affiliations:** ^1^Department of Food & Biotechnology, Hanseo University, Seosan 31962, Republic of Korea; ^2^College of Korean Medicine, Semyung University, Jecheon 27136, Republic of Korea

## Abstract

The aim of this study was to compare the effectiveness of velvet antler (VA) from different sections for promoting longitudinal bone growth in growing rats. VA was divided into upper (VAU), middle (VAM), and basal sections (VAB). An in vivo study was performed to examine the effect on longitudinal bone growth in adolescent rats. In addition, in vitro osteogenic activities were examined using osteoblastic MG-63 cells. VA promoted longitudinal bone growth and height of the growth plate in adolescent rats. Bone morphogenetic protein-2 (BMP-2) in growth plate of VA group was highly expressed compared with control. The anabolic effect of VA on bone was further supported by in vitro study. VA enhanced the proliferation, differentiation, and mineralization of MG-63 cells. The mRNA expressions of osteogenic genes such as collagen, alkaline phosphatase, and osteocalcin were increased by VA treatment. These effects of in vivo and in vitro study were decreased from upper to basal sections of VA. In conclusion, VA treatment promotes longitudinal bone growth in growing rats through enhanced BMP-2 expression, osteogenic activities, and bone matrix gene expressions. In addition, present study provides evidence for the regional differences in the effectiveness of velvet antler for longitudinal bone growth.

## 1. Introduction

Longitudinal bone growth, which determines the size and shape of the body frame, proceeds at a rate distinct from that of muscle and other tissues and is controlled by specific mechanisms. During childhood and adolescence, the long bones grow at the ends of the bones which occur at the growth plate by endochondral ossification of epiphyseal plate [1]. Growth plate chondrocyte proliferation and hypertrophy lead to formation of new cartilage, chondrogenesis, and remodeling of the newly formed cartilage into bone tissue resulting in longitudinal bone growth [2].

The current methods for increasing final height include treatment with a growth hormone alone or in combination with an analog of gonadotropin-releasing hormone [3]. However, it is generally too expensive and also has harmful side effects such as prepubertal gynecomastia, arthralgia, edema, benign intracranial hypertension, insulin resistance, and leukemia [4]. On that account, there has been an increasing awareness of the benefits of natural products, and many plant-derived medicines have been reported. However, the researches on animal medicines or functional foods are still relatively few, although animal medicines have been proven of various important components, such as proteins, peptides, fatty acids, glycosaminoglycans, prostaglandins, vitamins, minerals, dietary fiber, essential oils, and carotenoids [5, 6], which can be used in the prevention and treatment of various diseases.

Velvet antler (VA), the immature antler of male deer, is traditionally used for thousands of years in Asian countries, such as Korea, China, Taiwan, and Mongolia. It is currently estimated that the global production of velvet antlers is near to 1300 tons/year, which is still rapidly growing to meet the requirements of medicinal markets [7]. As a typical traditional animal medicine, VA has pharmacological effects to improve immune system, physical strength, and sexual function [7, 8]. VA, also, has been reported to possess bone strengthening activity and has been used in treating arthritis, osteoporosis, and fracture in animal model or human clinical trials [9–12].

A whole VA stick is usually divided into four portions, with the value decreasing from the tip to the base. Chemical analyses of VA revealed that there are regional differences in chemical composition: the contents of proteins and lipids decrease downward from the tip to the base, while those of ash, calcium, and collagen increase [13] suggesting the degree of calcification increased from tip to base section of VA. During the ossification of deer antler, the total collagen content was found to be increased. Typically, the antler is cut off near the base after it is about two-thirds of its potential full size, between 55 and 65 days of growth, before any significant calcification occurs. Traditionally, the market values of antlers are downgraded with increasing degree of calcification. However, which parts of VA are suitable for preventing and managing bone health especially bone growth had not been clarified.

The objective of the present study was to compare the effect of VA from different sections on longitudinal bone growth of adolescent rats and elucidate the underlying mechanisms for the effect.

## 2. Materials and Methods

### 2.1. Materials

**Figure 1:**
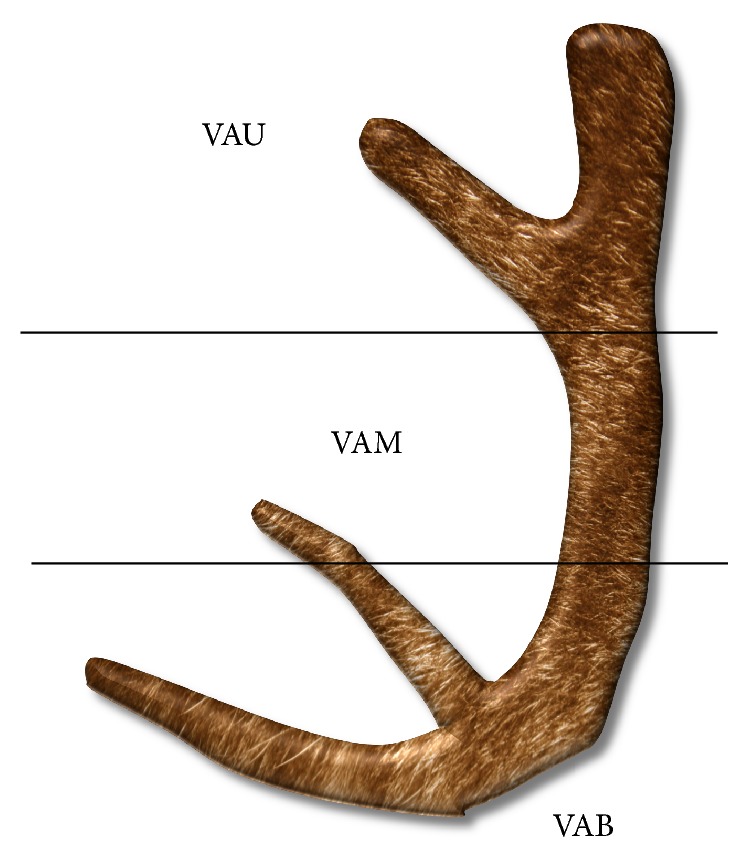
Sections of velvet antler. Fresh velvet antler was divided into upper (VAU), middle (VAM), and basal (VAB) sections.

VA, obtained from farmed Elk deer 75 days after casting, was kindly provided by the Animal Genetic Resources Station (National Institute of Animal Science, South Korea). VA was divided into upper (VAU), middle (VAM), and basal (VAB) sections (Figure 1). The criteria used for dividing the antler into three sections is defined as follows: upper section, top 15 cm of the part of the main beam to the 2nd division of a deer's antlers from its head; middle section, the upper half of the remaining main beam; and base section, the lower half of the remaining main beam. Total part of each section was sliced with a bone slicer, freeze-dried, and ground into powder. The powdered VA was immersed in 70% ethanol for 3 days, filtered, concentrated by vacuum evaporation, and finally subjected to freeze drying. Respective yields of sections, from upper to basal, were 3.4% (VAU), 2.9% (VAM), and 1.7% (VAB).

### 2.2. Animals

Longitudinal bone growth was determined in 3-week-old male Sprague-Dawley rats (Samtako Co., Osan, Korea). The experimental procedures were performed in accordance with protocols approved by the Institutional Animal Care and Use Committee, Semyung University (smecae 15-09-01). The animals were housed under controlled temperature and lighting conditions. The rats were randomly divided into four groups (*n* = 8); vehicle (control, distilled water) and upper (VAU), middle (VAM), and basal (VAB) sections of antler extract (100 mg/kg) were orally administered daily via stomach tube for 5 consecutive days. Treatment dose was decided from the preliminary study. On the sixth day, all rats were sacrificed under chloral hydrate anesthesia for tissue analysis.

### 2.3. Measurement of Longitudinal Bone Growth

To measure the effect on longitudinal bone growth rate, calcein was used as a fluorescence marker to label the bone line on the surface of the tibia. Calcein plays the role of fluorescent dye under ultraviolet illumination. Calcein (10 mg/kg, Sigma-Aldrich, St. Louis, MO, USA) was injected intraperitoneally 24 h before sacrifice. The dissected tibias were fixed in 0.1 M phosphate buffered formalin for 2 h, decalcified, and dehydrated by immersing in 30% sucrose for 1 day at 4°C. Dehydrated bone was sectioned longitudinally at sagittal sections of proximal part with a thickness of 40 *μ*m using a sliding microtome (HM440E, Zeiss, Germany). Bone growth was measured by measuring the gap between fluorescent line formed by calcein and the epiphyseal end line of the growth plate at three different locations using a fluorescent microscope (BX60, Olympus, Tokyo, Japan), and the averages were obtained.

### 2.4. Measurement of Growth Plate Height

Cresyl violet staining (Sigma-Aldrich) was used to stain the chondrocytes in the growth plate of the samples. Each tibia sample was sectioned longitudinally at a thickness of 40 *μ*m using a sliding microtome as described above and stained with cresyl violet. The growth plate height was measured at three different locations and the averages were obtained.

### 2.5. Bone Morphogenetic Protein-2 Expression

For the detection of bone morphogenetic protein-2 (BMP-2) in the growth plate, the dehydrated tibia sections were incubated overnight in 1% Triton X-100 containing goat BMP-2 antibody (Santa Cruz Biotechnology, diluted 1 : 500) at room temperature. Then, sections were incubated with anti-goat antibody (Vector Laboratories, Burlingame, CA, USA, diluted 1 : 200) for 60 min and stained with 0.05% 3,3-diaminobenzidine (Sigma Chemical Co.) containing 0.03% hydrogen peroxide.

### 2.6. Cell Culture

Human osteoblast-like MG-63 cells were obtained from the American Type Culture Collection (Rockville, MD, USA). Cells were cultured at 37°C in a 5% CO_2_ atmosphere in Dulbecco's Modified Eagle's Medium (DMEM, Gibco, MD, USA) containing 10% heat-inactivated FBS (Invitrogen, Grand island, NY, USA) and 100 U/mL penicillin/streptomycin (Sigma-Aldrich). Bone marrow cells prepared from the femur of male ICR mice were cultured in *α*-modified minimal essential medium (*α*-MEM, Gibco) containing 10% heat-inactivated FBS. After 3 days of culture, floating cells were removed and attached cells were used as osteoclast precursors.

### 2.7. Osteoblasts Proliferation

The effects of VA from different sections on proliferation of MG-63 cell were determined by a colorimetric immunoassay kit (Roche Diagnostics, Mannheim, Germany) which is based on quantitating bromodeoxyuridine (BrdU) incorporation into the newly synthesized DNA of replicating cells. MG-63 cells were seeded (5,000 cells/well) in 96-well plates containing DMEM with 10% FBS and allowed to adhere overnight. The media were replaced with DMEM containing VA samples (10, 50, and 100 *μ*g/mL) and incubated for 24 h. Subsequently, BrdU was added to each well and reincubated for 2 h. After 14 h of incubation at 37°C, labeling media was removed, cells were fixed, and the cells with BrdU label in the DNA were located with peroxidase-conjugated anti-BrdU antibody solution. Then bound anti-BrdU with substrate was colorimetrically measured with a microplate reader (BioTek Inc., Winooski, VT, USA) at 450 nm.

### 2.8. Alkaline Phosphatase Activity

Alkaline phosphatase (ALP) activity was measured using enzymatic assay. MG-63 cells, incubated in DMEM, were seeded in 12-well plates (50,000 cells/well) and incubated for 24 h. The medium was changed to osteogenic medium (DMEM with 10 mM *β*-glycerophosphate, 5 nM dexamethasone, and 50 *μ*g/mL ascorbic acid); VA samples (50 and 100 *μ*g/mL) were added to the cells and incubated for another 24 h. The 50 and 100 *μ*g/mL concentrations were chosen since the maximum effect and plateau had been gained under those two concentrations in cell proliferation assay results. The cells were then rinsed with PBS and lysed in 300 *μ*L of 0.2% of Triton X-100. The cell lysates were centrifuged at 13,000 rpm for 5 min. The supernatant of the lysate was used for the measurement of ALP activity by measuring the release of* p*-nitrophenol from* p*-nitrophenylphosphate at pH 9.8.

### 2.9. Collagen Content

Collagen synthesis was measured using picrosirius red method [14]. MG-63 cells were seeded in 12-well plates (50,000 cells/well) and allowed to adhere overnight. The medium was changed to osteogenic medium; VA samples (100 *μ*g/mL) were added to each well and incubated for 7 days. Cells were fixed with Bouin's fluid and stained with 0.1% Sirius red (Direct Red 80, Sigma-Aldrich) in a saturated aqueous solution of picric acid for 30 min. Stained dye was dissolved and the absorbance was measured at 540 nm.

### 2.10. Calcium Deposition

The formation of calcium phosphate was determined by alizarin red-S assay [15]. MG-63 cells were seeded and incubated as described above in the collagen assay process. After 7 days, cells were fixed with 10% formaldehyde and stained with 2% of alizarin red-S (pH 4.2, Sigma-Aldrich) at room temperature. The stained alizarin red-S was extracted with 10% acetic acid, and the amount of calcium deposition was quantified using the absorbance of extracted alizarin red-S at 405 nm.

### 2.11. Real-Time PCR

Real-time PCR was performed to analyze relative gene expression. MG-63 cells were seeded in 6-well plates (100,000 cells/well) and allowed to adhere overnight. The medium was changed to osteogenic medium; VA sample (100 *μ*g/mL) was added to each well and incubated for 24 h. Total RNA was extracted using RNeasy® Protect Mini Kit (Qiagen, Valencia, CA, USA), and cDNA was synthesized from mRNA using QuantiTect® Reverse Transcription Kit (Qiagen). Real-time PCR was performed using QuantiTect*™* SYBR® Green PCR Kit (Qiagen) according to the manufacturer's protocol. The PCR primer sequences are shown in Table 1. Analyses were performed using Rotor-Gene Q® (Qiagen) and gene expression values were calculated based on the comparative ΔΔCT method according to the manufacturer's protocol.

### 2.12. Osteoclastogenesis Assay

For induction of osteoclastogenesis, osteoclast precursor cells prepared as described above were further cultured for 4 days with RANKL (50 ng/mL) and M-CSF (30 ng/mL) in the presence of VA samples. Osteoclasts were identified by staining tartrate-resistant acid phosphatase (TRAP), a marker enzyme of osteoclasts [16], using commercial kit (TRAP 387-A Kit, Sigma-Aldrich). TRAP-positive multinucleated cells with ≥3 nuclei were counted as osteoclasts.

### 2.13. Statistical Analysis

The data were expressed as mean ± SD. One-way ANOVA followed by Tukey's multiple comparison test was performed for statistical analysis (GraphPad Prism ver. 6), and *P* values of less than 0.05 (*P* < 0.05) indicated significant differences. All in vitro experiments were performed with triplicate independent samples.

## 3. Results and Discussion

### 3.1. Effects on Cell Longitudinal Bone Growth

**Figure 2:**
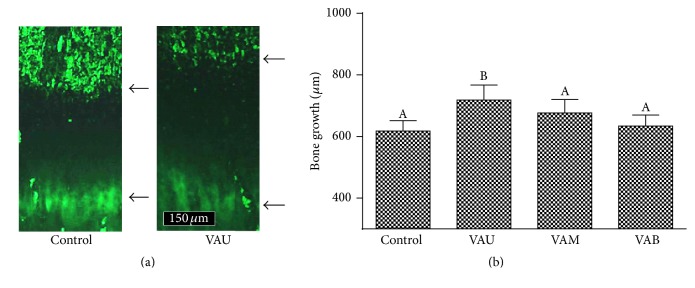
Effects of velvet antler on longitudinal bone growth. (a) Fluorescence photomicrographs of longitudinal sections of the proximal tibias of control and VAU (100 mg/kg) treated rats. (b) Numerical values of longitudinal bone growth. The arrow between the fluorescent line formed by calcein (lower arrow) and the epiphyseal end line of the growth plate (upper arrow) indicates the bone growth during the 24 h period. VAU: upper section of VA; VAM: middle section of VA; VAB: basal section of VA. The data are expressed as means ± SD. The bars with a different letter are significantly different from each other at the level of *P* < 0.05.

To determine the growth per day, instead of the total growth over a period, exact methods for measurement are necessary. Recently, the use of tetracycline or calcein as intravital markers of the growth process to label mineralizing bone in the rat has been reported [17, 18]. In the present study, calcein was used to label newly formed bone for the determination of longitudinal bone growth. Calcein binds to free calcium and gets deposited in newly deposited bone, causing staining and fluorescence under ultraviolet illumination. The effects of the different sections of VA on the rate of longitudinal bone growth from the proximal tibia in the rat are depicted in Figure 2. The fluorescent line corresponds to the injection of calcein which binds with calcium in the newly formed bone (Figure 2(a), lower arrow). The arrow between the fluorescent line formed by calcein (Figure 2(a), lower arrow) and epiphyseal end line of the growth plate (Figure 2(a), upper arrow) indicates the length of the bone growth during 24 h period. Longitudinal bone growth was significantly increased by VAU treatment compared with the control (Figure 2(a)).  Figure 2(b) shows the numerical values of the longitudinal bone growth. Longitudinal bone growth in control rat was 617.4 ± 34.2 *μ*m/day, and administration of VAU significantly increased the longitudinal bone growth to 718.6 ± 48.6 *μ*m/day (*P* < 0.05). VAU, VAM, and VAB caused 16.4%, 9.6%, and 2.7% increase in longitudinal bone growth, respectively, compared with control, suggesting that the effect decreases downward from upper section to the base. Although VAM caused slight increase in longitudinal bone growth, statistical significance was not observed. The present study provides first evidence for the effectiveness as well as regional differences of VA on longitudinal bone growth in adolescent rats.

Recently, Tseng et al. [19] compared the antiosteoporotic activity of VA from different sections using ovariectomy-induced osteoporosis animal model and suggest that the upper and middle sections of VA were equally effective in protecting bones from an estrogen-deficient state. However, the effects on proliferation and mineralization of osteoblasts, MC3T3-E1 cells, were decreased downward from upper section to the base [19] supporting the results of the present study. In addition they reported that the level of insulin-like growth factor 1 (IGF-1) was decreased downward from upper section to base section. Longitudinal bone growth is a function of growth plate chondrocyte proliferation and hypertrophy, and IGF-1 is reputed to augment longitudinal bone growth by stimulating growth plate chondrocyte proliferation [20]. Therefore, these data support the results of the present study, and one of the possible mechanisms may be explained by the IGF-1 level in each section of VA.

### 3.2. Effects on Growth Plate Heights

**Figure 3:**
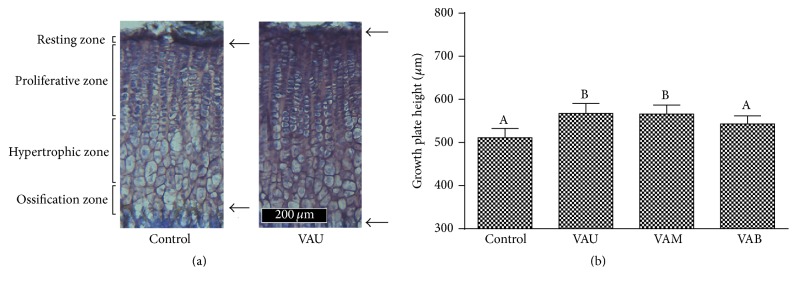
Effects of velvet antler on growth plate height. (a) Cresyl violet stained sections of tibial growth plates of control and VAU (100 mg/kg) treated rats. (b) Numerical values of growth plate height. Upper arrow indicates the resting zone where quiescent chondrocytes are waiting for the proliferation of the growth plates, and lower arrow indicates the ossification zone where cartilaginous matrix begins to calcify and replace with mineralized bone tissue. VAU: upper section of VA; VAM: middle section of VA; VAB: basal section of VA. The data are expressed as means ± SD. Values not sharing a common alphabet are significantly different from each other at the level of *P* < 0.05.

The growth plate, which is located at the distal end of the bone, is the main location where longitudinal bone growth occurs owing to the stimulation of chondrocyte proliferation. The growth plate consists of four distinctive histological zones beginning with the resting zone and extending through the proliferative, hypertrophic, and ossification zones (Figure 3(a)). The topmost layer, the resting zone, contains chondrocytes that serve as stem-like cells for the growth plate, with the potential to generate clones of rapidly proliferating chondrocytes, which are located in the proliferative zone in the second layer of the growth plate [21]. The proliferative zone is the driving force behind bone elongation. Over time, as longitudinal growth proceeds, proliferative cells close to the hypertrophic zone undergo terminal differentiation. During this process, they stop proliferating and physically enlarge to become hypertrophic chondrocytes, composing the third, hypertrophic layer of the growth plate. In the ossification zone, the chondrocytes eventually die and are transformed into bone matrix where longitudinal bone growth occurred [22].

Since the synchronized processes of chondrocyte proliferation and cartilage ossification in growth plate lead to longitudinal bone growth [23], the rate of longitudinal bone growth is regulated by the rate of chondrocyte proliferation of the growth plate [24]. In the present study, the rate of chondrocyte proliferation was determined by measuring the height of the proximal tibia growth plate (Figure 3(a)). The height of the growth plate in the control group was 511.2 ± 21.6 *μ*m, and administration of 100 mg/kg VAU and VAM significantly increased the height of the growth plate to 567.4 ± 23.4 *μ*m and 566.2 ± 20.6 *μ*m, respectively (Figure 3(b)). The effect of VAU (11.0%) was similar to that of VAM (10.8%), while VAB caused 6.3% increase which was not statistically significant compared with control. These results suggest that the effect of VAM was slightly lower but not statistically significant compared with VAU, and the effect decreases downward from upper section to the base.

### 3.3. Effects on BMP-2 Expression

BMPs play important roles in regulating growth plate chondrogenesis and longitudinal bone growth. Of the various forms of BMP, BMP-2 plays an important role in the development of the epiphyseal growth plate [25]. BMP-2 stimulates chondrocyte proliferation in the proliferative zone of the growth plate and also causes an increase in chondrocyte hypertrophy [26]. In this context, it has been suggested that alterations in the expression or production of BMP-2 can modulate the proliferation and activity of bone forming cells.

**Figure 4:**
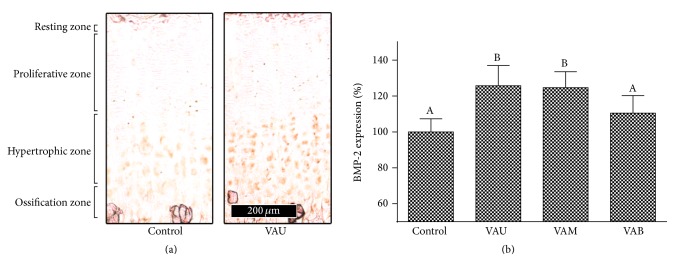
Effects of velvet antler on BMP-2 expression. (a) Immunohistochemical localizations of bone morphogenetic protein-2 (BMP-2) in the growth plates of control and VAU (100 mg/kg) treated rats. (b) Densitometric results of immunohistochemistry. VAU: upper section of VA; VAM: middle section of VA; VAB: basal section of VA. Data are shown as a percentage of the control value (means ± SD). Values not sharing a common alphabet are significantly different from each other at the level of *P* < 0.05.

In the present study, protein expression of BMP-2 in growth plate was highly expressed in hypertrophic and ossification zones compared to resting and proliferative zones (Figure 4(a)). As expected, BMP-2 expressions particularly in hypertrophic and ossification zones of the growth plates were increased in the VAU and VAM treated groups compared with the control group (Figures 4(a) and 4(b)). Numerical values of the BMP-2 expression were increased by 25.8%, 24.7%, and 10.5% with VAU, VAM, and VAB treatment, respectively, compared with control (Figure 4(b)). The effect on BMP-2 expression in growth plate, which is related to the bone growth and formation, was also decreased downward from upper section to the base. Furthermore, the effect of VAM was similar to VAU. The results of the in vivo experiment suggest that treatment with VA increases longitudinal bone growth rate by promoting chondrocyte proliferation and chondrogenesis through the upregulation of BMP-2 expression in growth plate, and the effect decreases downward from upper section to the base.

### 3.4. Effects on MG63 Cell Osteogenesis

**Figure 5:**
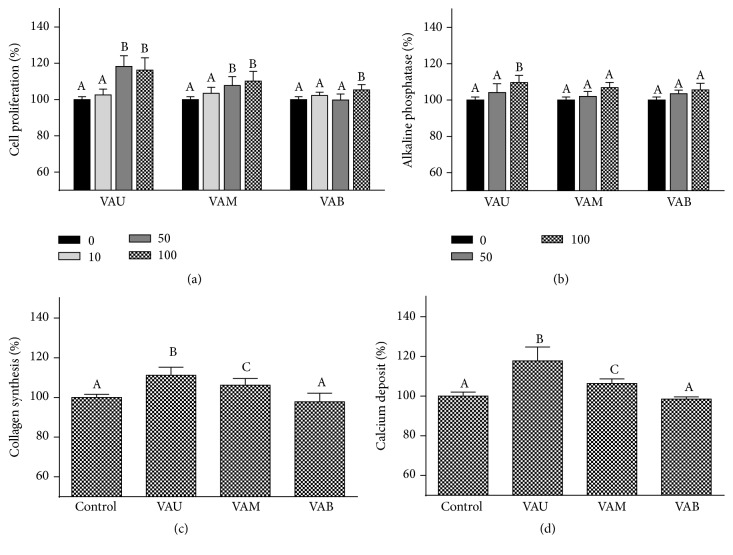
Effects of velvet antler on cell proliferation (a), alkaline phosphatase activity (b), collagen synthesis (c), and calcium deposit (d) in MG-63 human osteoblast-like cells. VAU: upper section of VA; VAM: middle section of VA; VAB: basal section of VA. Data are shown as a percentage of the control value (means ± SD) of the three cultures. Values not sharing a common alphabet are significantly different from each other at the level of *P* < 0.05.

Bone regeneration is regulated by a fine balance of biochemical and cellular events that ultimately stimulate osteoblasts to produce new tissue, in particular, new extracellular matrix composed mainly of collagen. The collagen matrix is then mineralized via ALP activity, which induces formation of calcium phosphate crystal seeds. Bellows et al. [27] reported that ALP is one of the osteoblast phenotype markers and an essential enzyme for mineralization. Hence, osteoblast proliferation, collagen content, and ALP activity, as early differentiation markers, and cellular calcium content, as a late marker of differentiation, were examined to investigate the effects of VA on the differentiation of MG-63 cells. First, the effect of different sections of VA on osteoblast proliferation was examined by BrdU incorporation. As shown in Figure 5(a), MG-63 cell proliferation was significantly and dose-dependently increased with VAU and VAM treatment (*P* < 0.05). MG-63 cell proliferation reached peak value at 50 *μ*g/mL VAU treatment exhibiting 119% of the basal value, while VAM treatment reached peak at 100 *μ*g/mL exhibiting 111% of the basal value. VAB at the highest concentration (100 *μ*g/mL) significantly (*P* < 0.05) increased cell proliferation compared to the control exhibiting 106% of the basal value. Effects of VAU and VAM on increasing cell proliferation were significantly higher than those of control at 50 and 100 *μ*g/mL concentration. Therefore, the effect of VA on ALP activity in MG-63 cells was further determined at 50 and 100 *μ*g/mL concentration. VAU treatment significantly increased the ALP activity, an early-stage osteoblasts differentiation marker, at 100 *μ*g/mL concentration (*P* < 0.05, Figure 5(b)) compared with control. VAM and VAB failed to affect ALP level. Hence, collagen content (Figure 5(c)) and calcium deposition (Figure 5(d)) were determined at 100*μ*g/mL of VA concentration. Collagen synthesis was significantly increased with VAU (11.2%) and VAM (6.2%) treatment compared with control, and the increased collagen synthesis was significantly higher than VAB. Cellular calcium deposition was examined using Alizarin red-S staining. Significant increases in mineralization were found with VAU and VAM treatment compared to the control and VAB group. Mineralization was significantly increased to 118% and 107% of the control by VAU and VAM treatment, respectively. The anabolic effect of VA on bone was further supported by the findings in this study demonstrating that VA enhanced the proliferation, differentiation, and mineralization of osteoblastic cells. Furthermore, the effects of VAU were greater than the effect of VAM, suggesting that the effects were decreased from the upper section to the base section. Lee et al. [28] reported that VA water extract enhanced osteoblasts proliferation and mineralization. Furthermore, Tseng et al. [19] reported that VAU and VAM dose-dependently increased osteoblasts proliferation as well as mineralization supporting the results of the present study.

### 3.5. Effects on Osteogenic Gene Expression

**Figure 6:**
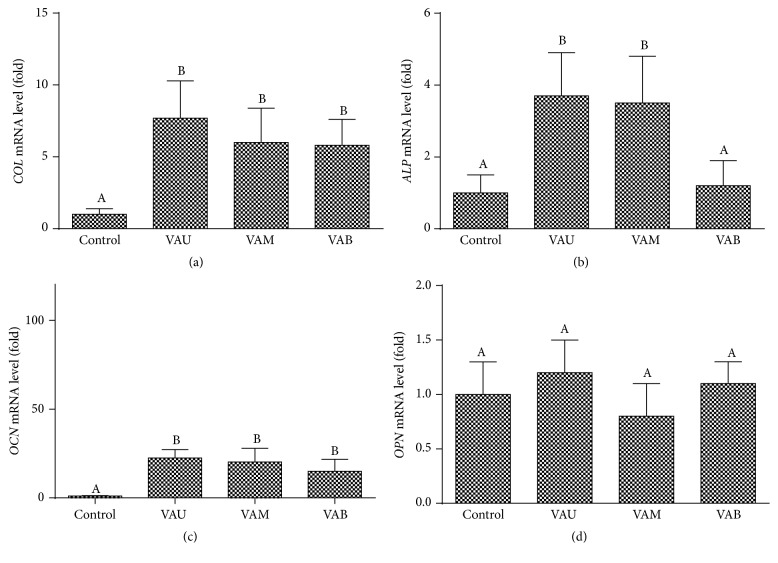
Effects of velvet antler on osteogenic gene expression in MG-63 cells. Real-time PCR was used to measure mRNA levels following VA treatment. Expression of GAPDH was used to normalize all samples. (a) Collagen (*COL*). (b) Alkaline phosphatase (*ALP*). (c) Osteocalcin (*OCN*). (d) Osteopontin (*OPN*). Data are shown as a fold of the control value (means ± SD) of the three cultures. Values not sharing a common alphabet are significantly different from each other at the level of *P* < 0.05.

It is known that preosteoblastic cells produce proteins of the extracellular matrix including collagen at first and then to successively produce alkaline phosphatase (ALP) and osteopontin/osteocalcin during mineralization phase [29]. Accordingly, there is increased expression of osteogenic genes in osteoblasts during the bone formation process, and these genes play roles in extracellular matrix formation and mineral deposition. Collagen (COL) is a primary gene product of osteoblasts during bone matrix formation and comprises 85–90% of the total organic bone matrix [30]. Noncollagenous matrix proteins also have a great importance in regulating of ossification and bone remodeling. The most abundant noncollagenous protein produced by osteoblasts is osteocalcin (OCN), a phosphorylated glycoprotein which has high affinity for binding ionic calcium and physiologic hydroxyapatite [31]. Osteopontin (OPN) is an osteoblast-derived, heavily glycosylated protein of the bone matrix, which is expressed at late stages of differentiation, and its appearance closely correlates with the appearance of mineral [31]. In order to examine the anabolic effect of VA, the mRNA expressions of* COL*,* ALP*,* OCN*, and* OPN* in MG-63 cells were measured by real-time PCR (Figure 6). All sections of the VA (VAU, VAM, and VAB) treatment caused marked upregulation of* COL* and* OCN* mRNA levels. Moreover, the effects of VAM and VAB were not significantly different from the effect of VAU in* COL* and* OCN* expressions (*P* < 0.05). The effects of VAU, VAM, and VAB on* COL* mRNA expressions were increased 7.7-, 6.0-, and 5.8-fold, respectively, while those of* OCN* expressions were increased 22.5-, 20.2-, and 15.0-fold, respectively. The expressions of* ALP* mRNA were significantly increased with VAU (3.7-fold) and VAM (3.5-fold) treatment compared with control and VAB group (1.2-fold).* OPN* mRNA expressions were not altered by all sections of VA. These data suggest that VA may play role at early stage as well as late stage of osteoblast differentiation, and VA-induced* COL*,* ALP*, and* OCN* gene expression could accelerate mineralization, subsequently leading to an increase in the extent of calcium deposition. Furthermore, the effects on osteogenic gene expressions were decreased from upper section to basal section of VA, and the effect of VAM was similar to VAU. Lee et al. [28] reported that VA increased mRNA expression of bone sialoprotein which is related to bone mineralization.

### 3.6. Effects on Osteoclastogenesis

Osteoclasts are the only cell type capable of resorbing mineralized bone. To examine the effect of VA on osteoclast differentiation, bone marrow-derived osteoclast precursor cells were cultured in osteoclastogenic media with different parts of VA extract. TRAP-positive multinuclear osteoclasts were generated in response to stimulation from M-CSF and RANKL. The results showed that the addition of VA did not affect osteoclast formation (data not shown).* Bacillus*-fermented VA extract has been reported to inhibit osteoclast differentiation [32] which does not support the results of the present study. However, Lee et al. [28] suggested that VA, fermented with* Cordyceps militaris*, contain more sialic acid than nonfermented VA, and the stimulatory effects on osteoblastic cell proliferation and ALP production were increased by fermentation. Therefore, the effect of nonfermented VA in the present study may not be strong enough to induce osteoclast differentiation.

## 4. Conclusions

In conclusion, results of the present study suggest that VA promotes longitudinal bone growth in adolescent rats through, at least in part, MG-63 cell osteogenesis, and the effect was decreased downward from upper section to basal section. VA stimulated the proliferation, differentiation, and mineralization of osteoblasts through upregulation of osteogenic gene expressions. These results support the stimulating nature of VA toward the function of osteoblastic cells. Although the molecular mechanisms underlying osteoblast differentiation remain to be defined, the results of the present study suggest that BMP-2, which plays an important role in regulating osteoblast differentiation and subsequent bone formation, might participate in these mechanisms. Furthermore, the present study provides strong evidence for the regional differences in the effectiveness of VA in longitudinal bone growth. However, further studies are needed to elucidate the bioactive chemical constituents associated with these effects.

The present study has some limitations. Only a single dose of VA was used in the in vivo study. The dose for traditional use of VA is usually 1 g/day, taken all at once or divided throughout the day. Therefore, the dose of 100 mg/kg/day given to the experimental rats in this study was equivalent to the traditional human dosing regimen of 1 g/day based on body surface area conversion, where animal dose is equal to human equivalent dose × 6.2, assuming that an adult person's weight is 60 kg [33].

## Figures and Tables

**Table 1:** Oligonucleotide sequences of osteogenic genes.

Primer	Direction	Sequence
Collagen (*COL*)	Forward	5′-GCG GCT CCC CAT TTT TAT ACC-3′
	Reverse	5′-GCT CTC CTC CCA TGT TAA ATA GCA-3′

Alkaline phosphatase (*ALP*)	Forward	5′-AAA CCG AGA TAC AAG CAC TCC CAC-3′
	Reverse	5′-TCC GTC ACG TTG TTC CTG TTC AG-3′

Osteocalcin (*OCN*)	Forward	5′-CCA TGA GAG CCC TCA CAC TCC TC-3′
	Reverse	5′-GCT TGG ACA CAA AGG CTG CAC-3′

Osteopontin (*OPN*)	Forward	5′-AGG CTG ATT CTG GAA GTT CTG AGG-3′
	Reverse	5′-GAC TTA CTT GGA AGG GYC TGT GGG-3′

*GAPDH*	Forward	5′-TCA TCA ATG GAA ATC CCA TCA CC-3′
	Reverse	5′-TGG ACT CCA CGA CGT ACT CAG C-3′
